# Yield stability of selected rice breeding lines and donors across conditions of mild to moderately severe drought stress

**DOI:** 10.1016/j.fcr.2016.09.011

**Published:** 2018-05-01

**Authors:** Rolando O. Torres, Amelia Henry

**Affiliations:** Crop and Environmental Sciences Division, International Rice Research Institute, Los Baños, Philippines

**Keywords:** Drought, Rice, Screening

## Abstract

Although mild to moderately severe drought stress may have less of an effect on rice grain yield than severe drought stress during reproductive stage, its prevalence across rice farmers’ fields at the global level may be more economically significant. In this study, field experiments were conducted on selected genotypes with known tolerance to severe reproductive-stage drought in order to identify those that would produce high and stable grain yield across seasons and soil moisture conditions varying from well-watered to mild and moderately severe drought stress. Mild stress generally occurred during wet seasons and moderate stress happened during dry seasons. The drought stress was mild enough such that the time to flowering was similar under drought stress and well-watered conditions in either season. However, significant grain yield reductions were incurred even at mild drought levels. Using an AMMI1 biplot analysis, IR83142-B-7-B-B, Binuhangin, IR77298-14-1-2-13, IR70215-70-CPA-3-4-1-3 and IR77298-14-1-2 were identified as the genotypes with the highest and most stable grain yields in both well-watered and mild to moderately severe drought stress environments. In a characterization of traits conferring drought tolerance among the highest yielding genotypes under mild to moderate drought stress, genotypes Binuhangin and IR70215-70-CPA-3-4-1-3 stood out for multiple physiological traits under drought. However, no direct correlations among genotypes between stomatal conductance, normalized difference vegetation index (NDVI) or root dry weight with grain or total dry matter yield were observed under any soil moisture level. These results reflect the complex interaction of drought response traits contributing to grain yield. The genotypic variation and physiological responses observed in this study point to the potential of developing varieties targeted to mild and moderate drought stress using yield as the selection criterion.

## 1 Introduction

Many different types of drought stress can affect rice crops, which can be characterized by soil moisture levels, the growth stages at which drought occurs, and the duration of the stress (Fukai and Cooper, 1995). Reduction in grain yield is particularly more serious if drought occurs during reproductive development (Hsiao and Namuco, 1980, Saini and Westgate, 2000, Pantuwan et al., 2002a), and severe yield losses can result even from a mild drought stress during the reproductive stage (O’Toole, 1982, Venuprasad et al., 2009, Verulkar et al., 2010). Thus, research studies have usually targeted drought stress at the reproductive stage. However, drought stress at any crop stage could reduce the grain yield to certain levels. Although the frequencies at which different types of drought stress occur in rice farmers’ fields on a global level have not been quantified, some general estimates based on average rainfall during the crop season indicate the importance of mild to moderate drought stress, particularly in Southeast Asia (Tsubo et al., 2006, Inthavong et al., 2011).

Mild to moderate drought stress usually occurs in rainfed and partially-irrigated fields when there is a lull in rainfall during the crop season. Studies on mild to moderate drought stress occurring at any stage or intermittently during the crop duration can be important for applicability, as this type of stress commonly occurs under actual farmers’ field conditions (Xangsayasane et al., 2014). Results from mild to moderate stress studies could be less prone to the high degree of variation that is typical of severe drought stress studies. Kumar et al. (2009) classified drought stress levels based on the relative yield reduction in which very severe, severe, moderate and mild stress is when the yield under drought stress is reduced by more than 85%, 60–85%, 40–60%, and less than 40% of the yield under non-stress conditions, respectively.

It is important to identify and develop genotypes that could produce high yields at a range of soil moisture conditions that actually occur in farmers’ rice fields. This study was conducted to evaluate the long-term performance of rice genotypes with the objective of identifying and selecting those with high and stable yields under well-watered and mild to moderate drought conditions across seasons of varying weather conditions. A subset of 10 genotypes was subsequently evaluated for physiological responses related to yield under the mild to moderate drought stress treatments.

## 2 Materials and methods

### 2.1 Screening for agronomic performance and yield stability

Experiments were conducted under well-watered (WW) and drought (DRT) conditions at IRRI Los Baños, Laguna, Philippines (14°30′N, 121°15′E). The soil belongs to the Maahas series which is classified as silty clay loam. Sixty genotypes (Table 1) were studied that had shown potential yield under drought in Genebank selection experiments (Torres et al., 2013) or had been used as parental donors in several IRRI research projects and breeding programs such as the Stress-Tolerant Rice for Africa and Southeast Asia (STRASA), International Network for Genetic Evaluation of Rice (INGER), IRRI Mini Genebank collections, and studies on QTL Lines and other Breeding Lines. The genotypes were grown during the wet season (WS) and dry season (DS) from 2008 DS – 2013 DS, for a total of 11 seasons with two environments (WW and DRT) per season. The entries that had comparatively low yields under drought stress during the first seven seasons of the experiment were replaced with new promising genotypes taken from some of the sources mentioned above. The genotypes and the durations when they were used in the experiments are listed in Table 1. Only the genotypes that were used until 2013 DS were included in the final statistical analysis.

**Table 1:** List of genotypes evaluated and duration over which they were planted in the screening experiments of this study.

Genotype	AMMI Code^a^	Seasons Planted		
		Start	End	Total
A05DS01-23	NA	08DS	08DS	1
A05DS01-26	NA	08DS	08DS	1
A05DS01-3	NA	08DS	08DS	1
Apo	G1	08DS	13DS	11
Aus196	G2	10DS	13DS	7
Binuhangin	G3	08WS	13DS	10
D6-446-6S	G4	08DS	13DS	11
D6-547-2R	G5	08DS	13DS	11
D6-547-5S	G6	08DS	13DS	11
Da8	G7	09DS	13DS	9
DGI-125	NA	08DS	09WS	4
DGI-138	NA	08DS	09WS	4
DGI-195	NA	08DS	09WS	4
DGI-196	G8	08DS	13DS	11
DGI-28	NA	08DS	10WS	6
DGI-32	G9	08DS	13DS	11
DGI-81	G10	08DS	13DS	11
DGI-81B	G11	10DS	13DS	7
DK106	G12	08DS	13DS	11
DK108	G13	08DS	13DS	11
DK109	G14	08DS	13DS	11
DK117	G15	08DS	13DS	11
DK122	G16	08DS	13DS	11
DK124	G17	08DS	13DS	11
DK135	G18	08DS	13DS	11
DK136	G19	08DS	13DS	11
DK142	G20	08DS	13DS	11
DK157	G21	08DS	13DS	11
DK167	G22	08DS	13DS	11
DK175	NA	08DS	08DS	1
DSL-69-6	G23	08DS	13DS	11
DSL-89-3	G24	08DS	13DS	11
DSU-18-6	NA	08DS	09WS	4
DSU-4-11	NA	08DS	09WS	4
DSU-4-18	G25	08DS	13DS	11
Dular	G26	10DS	13DS	7
FKR14	NA	08DS	08WS	2
HD1.4	NA	10DS	10WS	2
IR64	NA	08DS	08WS	2
IR71525-19-1-1	G28	08DS	13DS	11

Genotype	AMMI Code	Seasons Planted		
		Start	End	Total
IR70215-70-CPA-3-4-1-3	G27	08DS	13DS	11
IR71700-247-1-1	G29	08DS	13DS	11
IR74371-46-1-1	G30	08DS	13DS	11
IR74371-54-1-1	G31	08DS	13DS	11
IR75282-58-1-2-3	G32	08WS	13DS	10
IR75870-8-1-2-B-6-1-2-1-B	NA	08WS	09WS	3
IR75870-8-1-2-B-6-2-B-B-B	G33	08DS	13DS	11
IR75870-8-8-4-10-2-3-1	NA	08DS	10WS	6
IR75870-8-8-4-10-3-1-2	NA	08DS	08WS	2
IR77298-14-1-2	G34	08DS	13DS	11
IR77298-14-1-2-1	G35	08DS	13DS	11
IR77298-14-1-2-13	G36	08DS	13DS	11
IR77298-5-6-18	G37	08DS	13DS	11
IR77298-5-6-25	G38	08DS	13DS	11
IR78629-57-3-3-2	G39	11DS	13DS	5
IR78877-163-B-1-1	G40	08DS	13DS	11
IR78877-163-B-2-1	G41	11DS	13DS	5
IR78905-105-1-2-2	G42	08WS	13DS	10
IR78908-121-B-2-B	G43	08DS	13DS	11
IR78908-156-B-2-B	G44	08DS	13DS	11
IR78908-193-B-3-B	G45	08DS	13DS	11
IR78910-23-1-3-3	G46	08DS	13DS	11
IR78910-34-B-2-2	G47	08DS	13DS	11
IR79906-B-192-2-1	G48	08DS	13DS	11
IR81024-B-275-3-B	G49	08DS	13DS	11
IR81025-B-311-B	NA	08DS	08DS	1
IR81025-B-425-B	G50	08DS	13DS	11
IR83142-B-19-B-B	G51	11DS	13DS	5
IR83142-B-7-B-B	G52	11DS	13DS	5
IR83614-1007-B-B	G53	10DS	13DS	7
Jhum Sonalichikon	NA	09DS	09WS	2
Kalia	G54	09DS	13DS	9
M4FNS-2733	NA	08DS	10WS	6
M4FNS-3076	G55	08DS	13DS	11
N22	G56	10DS	13DS	7
Panama 1048	NA	08DS	08DS	1
PSBRc68	G57	08WS	13DS	10
RR72-18-832	G58	08DS	13DS	11
UPLRi7	G59	10DS	13DS	7
Uri	G60	09DS	13DS	9

‘NA’ indicates cultivars that were replaced from the trial and excluded in the biplot analysis.

a Genotype code used in the AMMI biplot analysis.

In each season, seedlings were raised on seed beds for 18 to 21 days before transplanting into the main experimental field. The experimental layout was generated using IRRIStat v. 5. An alpha lattice design was used with six blocks × 10 plots and 3 replications. The WW and DRT treatments were located in adjacent areas of the field that were separated by a permanent bund and a distance of about 5 m apart. The plots were 3 m long with 3 rows spaced at 25 cm between rows and 20 cm between plants within a row. Complete fertilizer was applied as basal at the rate of 40-40-40 kg NPK ha^−1^ and ammonium sulfate was topdressed during maximum tillering stage at the rate of 50 kg N ha^−1^ in both the WW and DRT treatments. The DRT treatment was initiated at four weeks after transplanting by withdrawing the irrigation supply and opening the drainage outlets.

Re-watering by surface flooding was done when the tensiometer readings were about −65 kPa at 30 cm soil depth. The WW treatment was kept continuously flooded with about 2 cm surface water until about ten days before harvest.

In the drought stress treatment, soil volumetric moisture content and soil matric potential at 30 cm depth were monitored using a Diviner 2000 (Sentek Sensor Technologies, Stepney SA, Australia) and dial-gauged tensiometer (Soilmoisture Equipment Corp., CA, USA), respectively. One Diviner 2000 observational tube and one tensiometer were installed in each replication after draining the DRT treatment plots when the soil dried to near field capacity. Readings from these devices were suspended when the soil was soaked or flooded after re-watering or when there was rainfall and resumed again when the soil was at about field capacity until the next drought episode. In 2012 WS, Diviner 2000 tubes and tensiometers were not installed because the field had been continuously soaked or flooded until maturity due to rainfall. Rainfall data were acquired from the IRRI agro-meteorological station located about 300 m from the experiments. The amount of rainfall that occurred from 50 to 110 DAS, which corresponds to the period from irrigation withdrawal in the DRT treatment to about hard dough stage of the grains, was considered as the effective rainfall for the drought-stressed crop.

The number of days to flowering (DTF) was recorded when at least 50% of the hills in the plot started to flower. Plant height was measured from ground level to the highest part of three random plants per plot at maturity. Above-ground biomass and grain yield at harvest were determined from the central 2 m of 3 rows per plot. Grain yield was normalized to 14% grain moisture content. Total dry matter yield was calculated as the sum of the oven-dry weights of above-ground parts normalized to 3% moisture content.

### 2.2 Physiological response of selected cultivars to drought

Ten entries that had shown high yield potential under drought in the initial screening experiments and genotype IR77298-5-6-B-11 that had been observed to be susceptible to drought (Swamy et al., 2013) were selected to characterize their physiological response to drought stress. The physiology study was conducted under WW and DRT conditions using a randomized complete block design with four replications in 2013 WS, 2014 DS, and 2014WS. The fields and experimental protocol for the agronomic practices and water treatments used were the same as those used in the preceding screening experiments. Physiological measurements on these 11 genotypes included canopy temperature (MI-210, Apogee Instruments, Logan UT, USA), stomatal conductance (AP4 porometer, Delta-T Devices, Cambridge, UK), and Normalized Difference Vegetation Index (NDVI, Greenseeker Hand-held Sensor, NTech Industries, CA, USA). NDVI is a measure of the density of green vegetation on a land area based on spectral reflectance and calculated as: (near infrared − red reflectance)/(near infrared reflectance + red reflectance). The canopy temperature, NDVI, and stomatal conductance observations were conducted only in the drought treatment at about mid-day during sunny days and when plants exhibited leaf rolling as a symptom of drought stress. Photosynthesis was measured at flowering during the drought period in the DS using a Li-Cor 6400 (Li-Cor Inc., Lincoln, Nebraska USA). Root samples were collected during flowering stage mid-way between 2 hills from 3 locations per plot in the 2014WS using a 4-cm diameter, 60-cm long soil core sampler. The soil core samples were sectioned into 15 cm lengths to determine the root distribution with depth to 60 cm. The grain and total dry matter yields were determined using the procedure described for the screening study.

### 2.3 Statistical analysis

The yield stability of the genotypes under drought and well-watered treatments across all 11 crop seasons and both treatments was determined with an additive main effects and multiplicative interaction (AMMI 1) biplot analysis using STAR Ver. 2.1 software. The AMMI 1 biplot allowed the identification of genotypes with both high and stable grain yields across varying soil moisture levels and seasonal environmental conditions, based on their proximity to the x-axis (PC1) and their mean grain yield. Genotypic variation in physiological traits was evaluated by ANOVA and LSD using the same STAR software and R v. 3.1.0 (R Foundation for Statistical Computing). Traits were correlated using linear regression in R v. 3.1.0.

## 3 Results

### 3.1 Rainfall and soil moisture conditions

**Fig. 1:**
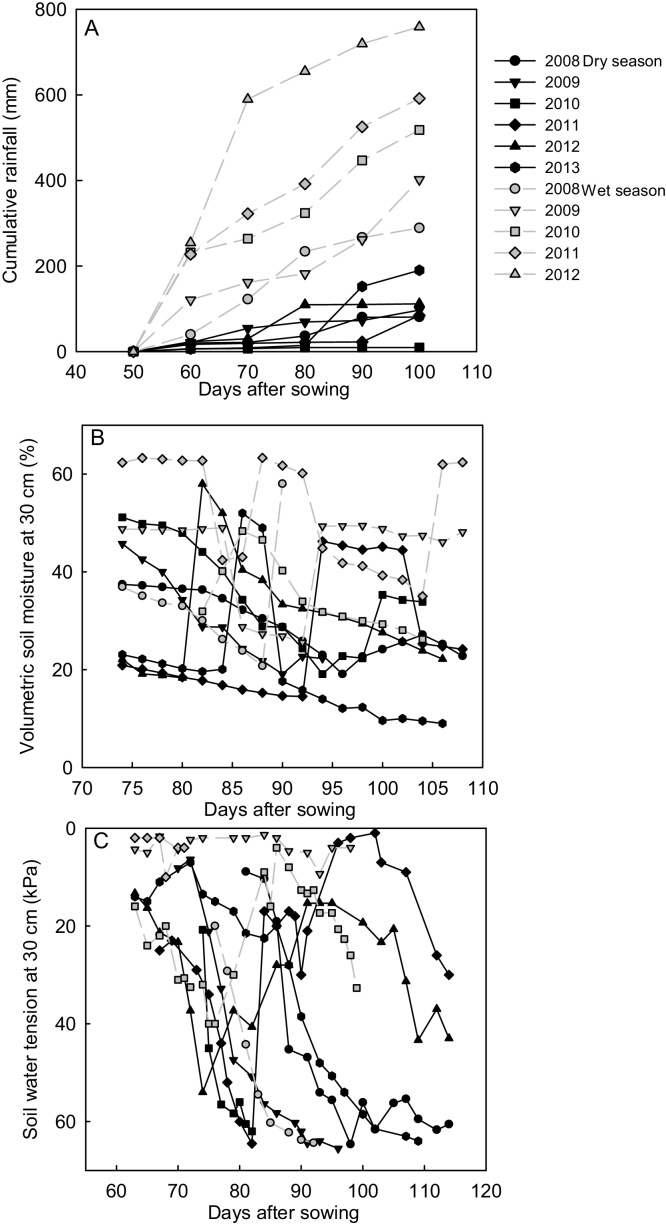
(A) Effective cumulative rainfall, (B) soil moisture content and (C) soil water tension at 30 cm depth in the dry and wet seasons each year during the drought stress period of the yield stability screening.

In the yield stability screening, the average effective rainfall that was received from four weeks after transplanting until about hard dough stage was 95 mm in the DS and 512 mm during WS (Fig. 1A). The total amount of effective rainfall during DS was highest in 2013 and lowest in 2010. The amount of rainfall varied widely during DS, whereas rainfall was more evenly distributed throughout the WS. The lowest soil moisture content (MC) in a season at the soil depth of 30 cm ranged from 9 to 19% during DS and 21 to 35% during WS (Fig. 1B). The highest soil water tension at 30 cm soil depth in the dry seasons exceeded 60 kPa, except in 2012 (Fig. 1C). The soil was driest during the reproductive stage until maturity especially in 2008, 2009, and 2013 dry seasons. During wet seasons, the maximum soil water tension incurred was 40 kPa, except in 2008 when it exceeded 60 kPa.

### 3.2 Agronomic response and yield stability of rice under varying drought levels

#### 3.2.1 Effect of drought on the growth and yield of rice across seasons

A total of 20 genotypes that showed very low biomass and grain yield under drought were replaced with more promising genotypes starting from 2008 DS until 2010 WS (Table 1). The mean number of days to reach 50% flowering was similar between the WW and DRT treatments except in 2009 WS, 2010 DS, and 2011 DS (Table 2). Drought reduced the mean plant height, total dry matter yield and harvest index by 19, 34, and 15%, respectively, during DS but the mean reductions during WS were less than 9% on any of these parameters. Mean plant height ranged from 70 to 126 cm and 91–134 under drought and well-watered conditions, respectively. Mean grain yield compared to the WW treatment declined by about 44% and 8% during DS and WS, respectively, in the DRT treatments. The highest grain yield reduction was 63.5% which occurred in 2008 DS. In all other seasons the yields in the DRT treatments were reduced by less than 50%. There were no significant drought effects on grain yield in the 2011 and 2012 wet seasons.

**Table 2:** Mean number of days to flowering (DTF), plant height (HT, cm), grain yield (GY, kg ha^−1^), total dry matter yield (TDMY, kg ha^−1^) and harvest index (HI, %) in the drought (DRT) and well-watered (WW) conditions during wet and dry seasons. Letters indicate difference significance groups at alpha = 0.05.

Season	Year	DTF		HT		GY		TDMY		HI	
		WW	DRT	WW	DRT	WW	DRT	WW	DRT	WW	DRT
Dry Season	2008DS	86.2a	83.9a	91a	70b	4731a	1729b	9413a	4591b	50.7a	37.9a
	2009DS	80.5a	80.1a	115a	93b	5337a	3122b	10203a	7936a	46.3a	34.8b
	2010DS	72.9a	76.9b	98a	80b	4964a	2680b	10124a	7525b	43.3a	31.2b
	2011DS	71.2a	74.9b	98a	81b	3579a	1818b	8624a	4448b	36.5a	36.0a
	2012DS	71.1a	71.4a	95a	73b	3254a	1828b	7071a	4489b	45.7a	40.7a
	2013DS	76.1a	76.6a	104a	88b	3402a	2775b	9559a	7856b	31.6a	31.2a
	Mean	76.3	77.3	100	81	4211	2325	9166	6141	42.3	35.3

Wet Season	2008WS	78.5a	82.3a	96a	81b	2962a	2343b	9551a	7526b	30.8a	31.1a
	2009WS	81.1a	79.4b	122a	100b	3752a	3279b	9363a	8409a	35.9a	35.0a
	2010WS	75.2a	76.6a	134a	126a	3599a	3197b	9510a	9378a	33.9a	30.0a
	2011WS	75.1a	74.7a	113a	117b	2900a	2965a	8247a	7917a	32.0a	33.5a
	2012WS	76.7a	76.6a	121a	126a	3430a	3197a	9220a	9417a	38.7a	30a
	Mean	77.3	77.9	117	109	3329	3078	9178	8674	34.3	33.1

**Fig. 2:**
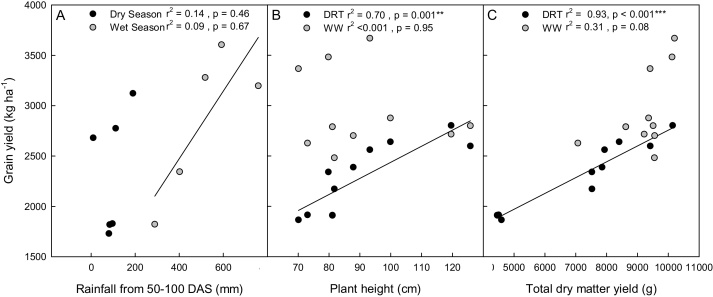
Relationships with grain yield and (A) effective rainfall (the total amount of rainfall from 50 to 100 days after sowing (DAS), (B) plant height, and (C) total dry matter yield (TDMY). Each point represents the mean value of one genotype.

Grain yield increased with increasing amount of effective rainfall in the wet seasons but there was no such trend in the dry seasons (Fig. 2A). Grain yield increased with increasing plant height in the DRT treatment but not in the WW treatment (Fig. 2B). Similarly, grain yield increased with increasing total dry matter yield (Fig. 2C) only in the DRT treatment.

#### 3.2.2 Grain yield production and stability of the genotypes under varying seasonal and moisture conditions

The top quartiles of the test genotypes in terms of mean grain yield over the total duration of the experiments under well-watered and drought treatments are shown in Table 3a. In the WW treatment, Apo, PSBRc68 and DSL-89-3 were the only genotypes that exceeded 4500 kg ha^−1^ mean grain yield across all seasons. In the DRT treatment, only IR83142-B-7-B-B, Binuhangin, IR83142-B-19-B-B, IR78877-163-B-2-1, and DK109 yielded more than 3000 kg ha^−1^. Genotypes DSL-89-3, DK167, DK109, IR83142-B-7-B-B, DK135, IR77298-14-1-2, Binuhangin, and IR70215-70-CPA-3-4-1-3 all appeared in the top quartile for yield in both WW and DRT treatments. Highest yields were obtained from IR83142-B-7-B-B, IR77298-14-1-2-13 and Apo in the wet season and from Binuhangin, Apo and PSBRc 68 in the dry season (Table 3b). There were 11 genotypes that were common in the upper quartile in grain yield of both wet and dry seasons.

**Table 3a:** Top quartile in grain yield (GY, kg ha^−1^) rank of the genotypes under well-watered and drought treatments across all crop seasons.

GY Rank	Well-watered			Drought		
	Genotype	GY	se	Genotype	GY	se
1	Apo	4668	221	IR83142-B-7-B-B	3224	201
2	PSBRc68	4593	229	Binuhangin	3194	136
3	DSL-89-3	4537	164	IR83142-B-19-B-B	3087	170
4	DK167	4475	237	IR78877-163-B-2-1	3081	239
5	D6-547-2R	4434	154	DK109	3053	268
6	DK109	4418	203	DGI-81	2892	197
7	IR83142-B-7-B-B	4335	254	DK167	2846	244
8	DK135	4332	147	DK135	2831	199
9	IR77298-14-1-2	4313	162	IR78629-57-3-3-2	2827	264
10	Binuhangin	4275	198	DK108	2815	184
11	IR77298-14-1-2-13	4253	227	Kalia	2803	175
12	DK124	4195	222	IR77298-14-1-2	2796	148
13	IR70215-70-CPA-3-4-1-3	4182	239	IR78877-163-B-1-1	2773	241
14	M4FNS-3076	4178	170	IR70215-70-CPA-3-4-1-3	2745	202
15	IR75870-8-1-2-B-6-2-B-B-B	4156	202	DSL-89-3	2739	172

**Table 3b:** Top quartile in grain yield (GY, kg ha^−1^) rank of the genotypes during the wet and dry seasons.

GY Rank	Wet Season			Dry Season		
	Genotype	GY	se	Genotype	GY	se
1	IR83142-B-7-B-B	3891	154	Binuhangin	3852	221
2	IR77298-14-1-2-13	3741	197	Apo	3849	300
3	Apo	3661	161	PSBRc68	3741	310
4	DK109	3647	92	IR83142-B-7-B-B	3740	241
5	DSL-89-3	3646	116	DK109	3633	281
6	IR83142-B-19-B-B	3620	117	IR77298-14-1-2	3611	243
7	DK135	3602	175	DSL-89-3	3610	264
8	Binuhangin	3559	128	DK167	3576	304
9	DK167	3536	139	IR83142-B-19-B-B	3562	226
10	IR77298-14-1-2	3527	185	DK135	3534	254
11	D6-547-2R	3525	105	DGI-81	3508	244
12	DGI-81	3515	174	D6-446-6S	3478	208
13	IR78877-163-B-2-1	3483	145	IR70215x	3463	290
14	IR75870x	3469	199	DK108	3449	242
15	DK142	3467	120	D6-547-2R	3417	274

**Fig. 3:**
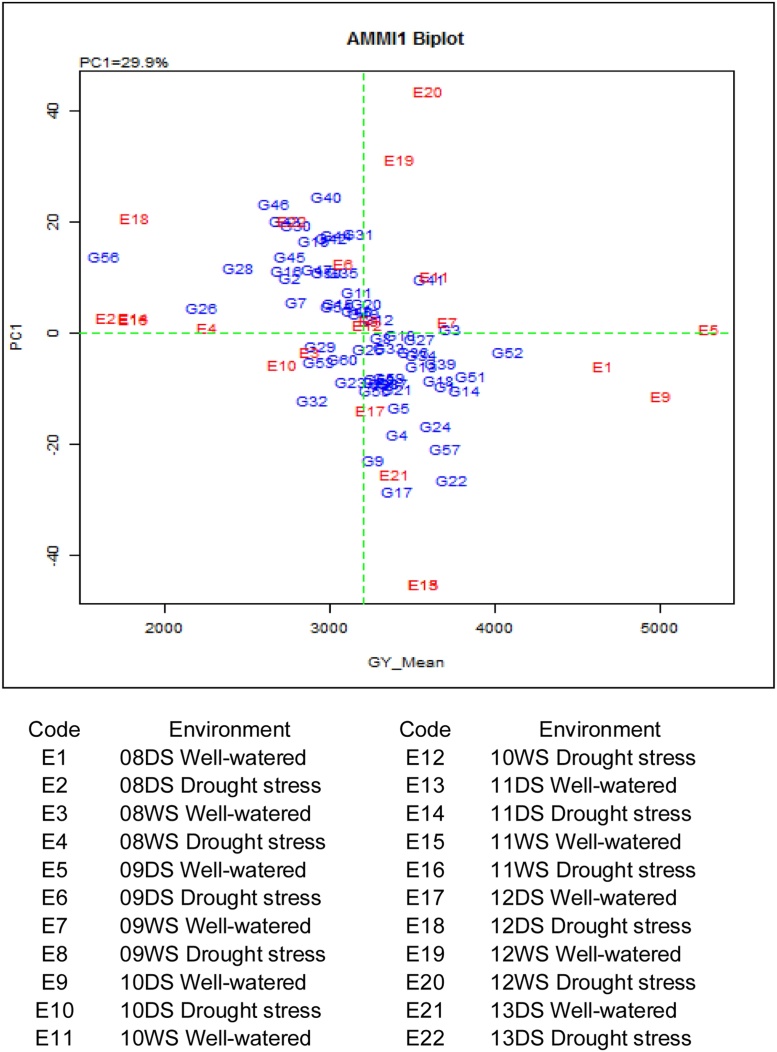
Compilation of grain yield data across 11 seasons and two treatments: AMMI biplot analysis of the first principle component and mean grain yield under drought and well-watered treatments. Genotype codes are listed in Table 1.

Based on the AMMI1 biplot analysis (Fig. 3), IR83142-B-7-B-B, Binuhangin, IR77298-14-1-2-13, IR70215-70-CPA-3-4-1-3, and IR77298-14-1-2 were the genotypes with the highest and most stable grain yields across all seasons including WW and DRT treatments.

### 3.3 Physiological response of the selected genotypes to drought

**Fig. 4:**
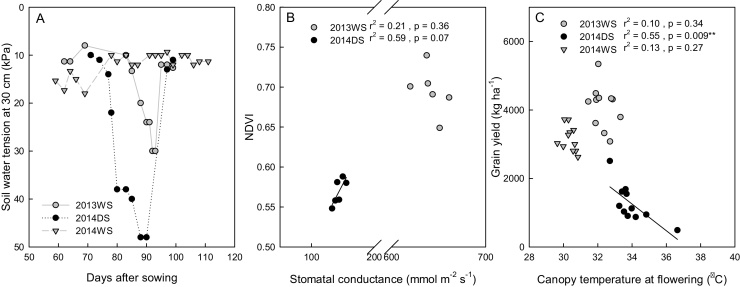
Key results from the physiology trials: (A) Soil moisture tension at different seasons after withdrawing the irrigation water in the physiological study, (B) the relationship between NDVI and stomatal conductance at flowering in the wet and dry season, and (C) the effect of canopy temperature at flowering on grain yield under drought. Each point in B and C represents the mean value of one genotype.

In the study on physiological response to drought, mild to moderate drought stress conditions were incurred as evidenced by the soil water tension records which did not exceed 30 kPa in the wet seasons and reached only 48 kPa in the dry season (Fig. 4A). In 2014 WS, the soil remained soaked to flooded and the only measurement conducted was root sampling. Plant height and total dry matter yield (Table 4) were reduced by drought more severely in the dry season than in the wet season. The number of tillers was not significantly affected by drought but the start of flowering was delayed by 9 days in the dry season. The number of fertile panicles (those containing at least a single filled spikelet), grain yield and harvest index were significantly reduced by drought during dry season but not in the wet season.

**Table 4:** Mean plant height (Ht, cm), Tillers (number hill^−1^) and total dry matter yield (TDMY, kg ha^−1^) at flowering and at harvest, number of days to flowering (DTF), fertile panicles (FPan, number hill^−1^), grain yield (GY, kg ha^−1^), and harvest index (HI, %). Letters indicate difference significance groups at alpha = 0.05.

Season	Water	DTF	Ht	Tillers	TDMY	FPan	GY	HI
DS	DRT	95a	67b	15.4a	6119b	8.9b	1293b	21b
	WW	87b	96a	15.8a	10942a	13.5a	4633a	43a
WS	DRT	83a	103a	15.0a	8539b	12.3a	3434a	38a
	WW	8 a	107a	14.3a	9189a	13.1a	3637a	39a
Mean	Dry Season	91a	81b	15.6a	8531a	11.2a	2963b	32b
	Wet Season	82b	105a	14.6a	8864a	12.7a	3535a	39a

Stomatal conductance increased with increasing NDVI under drought stress during the dry season (Fig. 4B). There were no significant differences among genotypes in NDVI or stomatal conductance in both seasons, in photosynthesis rate in the dry season, or in canopy temperature or root dry weight from the 45–60 cm layer during the wet season (Table 5). However, there were significant differences in grain yield and total dry matter yield in both seasons and in harvest index during the wet season. Binuhangin had the highest grain yield in both seasons under the drought treatment. The mean canopy temperature at flowering was lower by 1.7°C while the mean NDVI and stomatal conductance were higher by about 20% and 78%, respectively during the wet season than that in the dry season. The mean grain yield decreased with increasing canopy temperature at flowering stage in the dry season but not in the wet season (Fig. 4C).

**Table 5:** Physiological characteristics of selected lines. The NDVI, stomatal conductance (mmol m^−2^ s^−1^), photosynthesis (PS, μmol m^−2^ s^−1^) canopy temperature (°C) and total root dry weight (mg) in the soil core under drought during flowering, and the corresponding grain yield (GY, kg ha^−1^), total dry matter yield (TDMY, kg ha^−1^), and harvest index (HI, %) in dry and wet seasons under drought stress treatment. Letters indicate difference significance groups at alpha = 0.05.

Season	Genotype	NDVI	Conduct.	PS	Canopy Temp	Root dry wt	GY	TDMY	HI
Wet	Apo	0.726a	638a		31.9a	21.05a	4586ab	12022b	33.3abcd
	Binuhangin	0.701a	620a		32.0a	14.92a	5166a	11780bc	40.3a
	DGI-81	0.691a	644a		31.9a	13.63a	4185abcd	9130de	41.7a
	DK109	0.737a	651a		32.7a	16.53a	3228e	8612e	31.5abcd
	DK124	0.722a	654a		32.9a	21.42a	4251abc	11249bcd	34.0abcd
	DSU-18-6	0.649a	651a		32.1a	14.86a	4252abc	9655cde	39.9ab
	IR70215-70-CPA-3-4-1-3	0.700a	653a		31.5a	25.72a	4399abc	12586ab	29.8cd
	IR77298-5-6-B-11	0.704a	639a		32.8a	16.37a	4229abc	9688de	39.6abc
	IR83142-B-19-B-B	0.740a	638a		31.9a	15.95a	3578cde	8786e	36.5abc
	IR83142-B-7-B-B	0.700a	643a		32.4a	20.05a	3235de	9649e	30.5bcd
	PSBRc68	0.688a	661a		33.3a	23.80a	3856bcde	13855a	24.3d
	WS Mean	0.705	645		32.3	18.6	4088	10,638	34.7

Dry	Apo	0.530a			33.7bcd		1547ab	6695ab	20.0a
	Binuhangin	0.580a	155.5a	3.37a	32.7d		2507a	7508a	28.1a
	DGI-81	0.548a	133.0a	3.48a	33.5cd		1029ab	6194ab	14.1a
	DK109	0.569a			33.4cd		1613ab	6244ab	20.3a
	DK124	0.554a			34.2bcd		1712ab	6009ab	11.4a
	DSU-18-6	0.558a	138.1a	5.31a	33.8bcd		904ab	6564ab	12.1a
	IR70215-70-CPA-3-4-1-3	0.546a			33.6cd		1684ab	5918ab	24.1a
	IR77298-5-6-B-11	0.559a	144.1a	4.34a	34.8abc		943ab	5721ab	13.6a
	IR83142-B-19-B-B	0.588a	150.0a	2.67a	34.0bcd		1120ab	5259ab	18.2a
	IR83142-B-7-B-B	0.568a			36.7abc		486b	3948b	10.1a
	PSBRc68	0.581a	141.2a	4.53a	33.3cd		1195ab	7956a	13.7a
	DS Mean	0.562	143.6		34.0		1264	6183	20.1

**Fig. 5:**
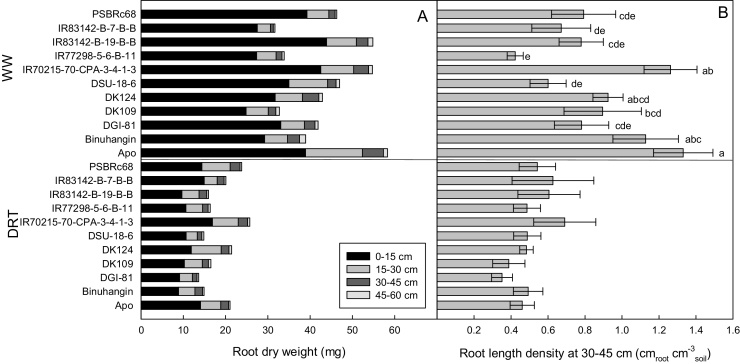
Root growth in the 2014 wet season under drought and well-watered treatments. (A) Root dry weight distribution in the 0–60 cm soil layer by 15-cm depth segments, and (B) the root length density from 30 to 45 cm. Letters indicate difference significance groups at alpha = 0.05.

In 2014WS, drought reduced the mean root dry weight of the genotypes within the 45–60 cm soil depth by 58% (Fig. 5A).The highest proportion of root dry weight was located in the upper 15 cm layer. Only 11.6% and 7.5% of the root dry weight was below the 30 cm soil depth in the DRT and WW treatment, respectively. Genotype IR70215-70-CPA-3-4-1-3 was one of the genotypes with significantly highest root length density at a depth of 30–45 cm in the WW treatment, and also showed relatively highest root length density at 30–45 cm in the DRT treatment (Fig. 5B). IR77298-5-6-B-11, which was included as a drought-susceptible genotype, showed comparable results with most genotypes for the parameters measured.

## 4 Discussion

### 4.1 Rainfall and soil moisture conditions

The range of rainfall from dry to wet season in this study resulted in a mild to moderate drought stress level in both the yield screening and physiological experiments. In the dry seasons, the drought stress was generally progressive, with an average rainfall of only about 95 mm available to the crop starting from about panicle initiation until maturity, although this rainfall was not evenly distributed. Drought stress was mild in the wet season where soil moisture tension was usually less that 40 kPa at the depth of 30 cm. These values are much lower than those observed under severe drought stress in IRRI lowland rice screening trials, which typically exceed 60 kPa at the depth of 30 cm (Henry et al., 2015, Swamy et al., 2013, Vikram et al., 2015).

### 4.2 Agronomic response and yield stability of rice under varying drought levels

#### 4.2.1 Effect of drought on crop growth across seasons

Flowering delay is a common effect of drought stress in rice (Zhao et al., 2010; Ndjiondjop et al., 2010, Pantuwan et al., 2002b), and has been suggested as a selection index based on the cumulative drought stress the rice crop experiences before heading (Homma et al., 2004). In this experiment, the drought stress was not severe enough to delay the onset of flowering, which was also probably due to the late initiation of drought stress. The relationship between flowering delay and grain yield has previously been reported to vary across sites and depending on the type of drought stress (Monkham et al., 2015). The stress severity was also reflected by reduction in growth and biomass production under drought, which was higher in the dry season than in wet season. Rainfall increased the grain yield in the drought stress treatment during the wet season but it was insufficient and was unevenly distributed during the dry season. The reduction in grain yield by drought was less than 50% in all but one of the 11 seasons, when it reached 63%. The differences in plant height and dry matter yield under drought that were not correlated with grain yield in the WW treatment demonstrate genetic drought-response characteristics. Under drought stress conditions, however, the genotypes that were able to grow better and produce higher biomass also produced higher grain yield, which is in agreement with Kumar et al. (2009) and Torres et al. (2013), who previously reported relationships between above-ground biomass and rice yield under drought.

#### 4.2.2 Grain yield production and stability of the genotypes under varying seasonal and soil moisture conditions

Some genotypes including Jhum Sonalichikon (Seshu and Garrity, 1986), DGI 125 and DSU 18-6 (Lafitte et al., 2007), and IR81025-B-311-B (Singh et al., 2013) that were previously observed to be drought resistant were eliminated because they did not compete well in this study; these were probably competitive only under severe drought stress where grain yield is not economically feasible. In contrast, IR77298-14-1-2 performed well in this study and was used as a parent of IR64-drought 1, which has been released as a variety for severe reproductive stage drought (A. Kumar, personal communication). These results suggest that some rice genotypes may show better adaptability to a range of drought severities, whereas other drought-tolerant genotypes may perform well under specific types of drought stress.

Different genotypes performed better in different seasons and soil water treatments; this variation necessitated a statistical approach integrating all trials to identify the best-performing genotypes across seasons and treatments (22 environments). AMMI analysis has previously been used to identify rice genotypes with stable yield across many variable environments (Raman et al., 2011, Anantha et al., 2016). The genotypes identified by the AMMI analysis as having high and stable grain yield in this study have been evaluated concomitantly in other studies; IR83142-B-7-B-B has shown good weed competitiveness (Chauhan et al., 2015); Binuhangin stood out for high yield under drought among diverse Genebank accessions (Torres et al., 2013); and IR77298 lines have exhibited low canopy temperature (Swamy et al., 2013); and despite showing promising performance here, IR70215-70-CPA-3-4-1-3 showed low pollen fertility in evaluation of genotypes for use in hybrid rice (Kamalnath Reddy et al., 2014).

### 4.3 Physiological response of selected cultivars to drought

In the physiology trials, mild drought occurred during the wet season due to rainfall. Stomatal conductance, canopy temperature and NDVI under drought were better correlated during the dry season than the wet season. The drought stress level may not have been severe enough to reflect the correlations between NDVI and root weight in their effect on grain yield. Despite these indications of mild stress in the wet seasons, there were significant yield reductions due to drought (Table 5). Of the stable and highest yielding genotypes identified by the AMMI analysis, two genotypes stood out in the physiology measurements. Binuhangin showed the highest NDVI, highest percentage of root weight below 30 cm, and lowest canopy temperature at flowering stage, had the highest grain yield and harvest index in the dry season drought stress treatments. IR70215-70-CPA-3-4-1-3 showed relatively lowest canopy temperature, high shoot biomass, and the highest root length density at the 30–45 cm depth under DRT and WW conditions. The lack of direct relationships between physiological traits and grain yield, as well as these combinations of traits in selected genotypes, indicate that different traits in combinations may be related to grain yield in each particular season.

## 5 Conclusions

Significant grain yield reductions were incurred even at the mild to moderate drought levels in this study. Genotypes IR83142-B-7-B-B, Binuhangin, IR77298-14-1-2-13, IR70215-70-CPA-3-4-1-3, and IR77298-14-1-2 showed the highest and most stable grain yields in both well-watered and drought environments across 11 seasons. Grain yield was most related to above-ground shoot biomass across genotypes, but selected genotypes identified for stable and high yield also stood out for multiple physiological traits. The genotypes identified as highest yielding under mild drought stress were not necessarily those that were previously highest yielding under more severe drought stress, which may have implications for breeding targets since mild drought stress likely affects a large proportion of drought-prone rice-growing areas.
